# Linking pulmonary function with rheumatoid arthritis

**DOI:** 10.6026/973206300214285

**Published:** 2025-12-15

**Authors:** Rahul Soni, Pankaj Kumar Gupta, Yasha Bandil, Ritu Rani Vinodia, Sachin Parmar

**Affiliations:** 1 Department of Pulmonary Medicine, BirsaMunda Government Medical College, Shahdol, Madhya Pradesh, India; 2 Departmentof General Medicine, BirsaMunda Government Medical College, Shahdol, Madhya Pradesh, India; 3 Department of Ophthalmology, BirsaMunda Government Medical College, Shahdol, Madhya Pradesh, India; 4 Department of ENT, BirsaMunda Government Medical College, Shahdol, Madhya Pradesh, India; 5 Department of Community Medicine, V.K.S.Govt. Medical College, Neemuch, Madhya Pradesh, India

**Keywords:** Rheumatoid arthritis, pulmonary function tests, restrictive lung disease

## Abstract

Rheumatoid arthritis (RA) is a chronic autoimmune disorder that frequently leads to pulmonary involvement, contributing to morbidity and mortality. Therefore, it is of interest to assess pulmonary function in 100 participants (50 RA patients and 50 controls) using spirometry. RA patients showed significantly higher odds of pulmonary function test (PFT) abnormalities, including FVC%, FEV1%, PEFR% and FEF 25-75%. A higher disease severity, longer disease duration and elevated serological markers (RF, ACPA, CRP) were strongly associated with these abnormalities. Routine spirometry should be incorporated into RA management for early detection and improved outcomes.

## Background:

Rheumatoid arthritis (RA) is the most prevalent inflammatory arthritis, impacting 1% of the general population [1]. Rheumatoid arthritis is a chronic multisystem disorder of unknown origin [2, 3]. The defining characteristic of established rheumatoid arthritis is persistent inflammatory synovitis, typically affecting peripheral joints in a symmetric pattern, despite various systemic manifestations. The capacity of synovial inflammation to induce cartilage damage, bone erosion and subsequent alterations in joint integrity is the defining characteristic of the disease. Notwithstanding its destructive capacity, the trajectory of RA can exhibit considerable variability. Some patients may encounter a mild oligoarticular condition of short duration with minimal joint damage, while the majority will suffer from a relentlessly progressive polyarthritis resulting in significant functional impairment [4, 5]. The disease is more prevalent in females than in males, with a female-to-male ratio of 3:1. The prevalence escalates with age and gender disparities diminish in the older demographic. The onset predominantly occurs in the fourth and fifth decades of life, with 80% of patients manifesting the disease between the ages of 35 and 50. The clinical manifestations of rheumatoid disease exhibit considerable variability, yet joint symptoms typically prevail. While acute presentations may arise; the emergence of articular inflammatory signs is typically insidious, accompanied by prodromal symptoms of nonspecific periarticular pain or stiffness. The hallmark manifestation of rheumatoid arthritis is pain, edema and rigidity of affected joints. Joint involvement is typically symmetrical. Symmetrical swelling of multiple joints accompanied by tenderness and pain is indicative of rheumatoid arthritis (RA) [5]. There is limited knowledge concerning the progression of these extra-articular manifestations. An atypical autoimmune reaction, genetic predispositions and certain environmental or biological influences, including viral factors Infection or hormonal fluctuations are recognized as triggers of rheumatoid arthritis (RA). Pleuro-pulmonary involvements and their manifestations represent one of the most prevalent extra-articular manifestations of rheumatoid arthritis and the second leading cause of death due to infection. Interstitial lung disease represents the most prevalent and severe manifestation of pulmonary involvement in rheumatoid arthritis. Radiographic alterations such as fibrosis and physiological changes, including restriction or diminished diffusing capacity on pulmonary function tests, may manifest in rheumatoid arthritis (RA) [6, 7- 8]. Although rheumatoid arthritis primarily causes restrictive lung disease, recent studies indicate that it can also lead to obstructive pulmonary disease. Therefore, it is of interest to assess pulmonary function test abnormalities in diagnosed cases of rheumatoid arthritis (RA) and to examine the correlation between lung function and RA disease activity.

## Methodology:

## Study design and setting:

This case-control study was conducted at a tertiary care hospital in India, enrolling 100 participants (50 RA patients and 50 age-matched healthy controls). Data were collected between January 2024 and December 2024.

## Participants:

[1] Cases: RA patients diagnosed using the 2010 ACR/EULAR classification criteria, aged 20-59 years, with no prior history of chronic lung disease, smoking, or systemic comorbidities affecting pulmonary function.

[2] Controls: Healthy individuals with no autoimmune or respiratory disorders, matched for age and gender.

## Data collection:

[1] Demographic and clinical data:

1) Age, gender and disease duration were recorded.

2) Disease activity was assessed using the Disease Activity Score-28 (DAS-28) and ACR/EULAR scores.

[2] Pulmonary function tests (PFTs):

1) Spirometry (COSMED PFT system) measured FVC%, FEV1%, PEFR% and FEF 25-75% following ATS/ERS guidelines.

2) Abnormal values were defined as <80% of predicted for FVC, FEV1 and PEFR and<60% for FEF 25-75%.

[3] Serological markers:

1) Rheumatoid factor (RF) and anti-citrullinated protein antibody (ACPA) levels were quantified via ELISA.

2) C-reactive protein (CRP) was measured using immunoturbidimetry (positive cutoff: ≥6 mg/L).

## Statistical analysis:

[1] Categorical variables (e.g., PFT abnormalities, seropositivity) were analyzed using Chi-square tests.

[2] Odds ratios (OR) with 95% confidence intervals (CI) quantified RA-lung dysfunction associations.

[3] p< 0.05 was considered statistically significant (SPSS v28.0).

## Ethical considerations:

Approval was obtained from the Institutional Ethics Committee and written informed consent was secured from all participants.

## Results:

The table compares demographic and pulmonary function test (PFT) parameters between 50 cases and 50 controls. Most cases are females (41 vs. 32 in controls) and predominantly in the 30-49 years age groups, whereas controls have a more even age distribution including more individuals aged 50-59 years. Regarding lung function, cases show a higher proportion of abnormal values across FVC%, FEV1%, PEFR% and especially FEF 25-75% predicted values compared to controls, indicating more impaired pulmonary function among cases. Controls mostly have normal predicted values, particularly for FVC% (49 normal vs. 28 cases) and FEV1% (37 normal vs. 28 cases), suggesting better lung function overall in the control group. Table 2 (see PDF) summarizes serological markers in 100 rheumatoid arthritis patients. Rheumatoid Factor was low positive in 62% and high positive in 38% of cases. ACPA levels were normal in 17%, low positive in 55% and high positive in 28% of patients. C - reactive protein (CRP) was positive in 67% and negative in 33% of cases. Table 3 (see PDF) presents the distribution of disease duration and ACR/EULAR scores among 100 rheumatoid arthritis cases. Most patients (60%) had disease duration of 3-4 years, followed by 23% with over 4 years and 17% with 1-2 years. According to ACR/EULAR scores, 96% had active disease (score 7-10), while only 4% were in the normal range (score 1-6). Table 4 (see PDF) displays the distribution of DAS-28 disease activity among 100 rheumatoid arthritis cases. None of the patients had inactive disease. Moderate disease activity was observed in 33% of cases; while the majority (67%) exhibited high disease activity. Table 5 (see PDF) highlights the association between rheumatoid arthritis and abnormal lung function parameters in 50 cases and 50 controls. RA cases showed significantly higher odds of abnormal FEF 25-75% (OR: 2.6, p=0.002), PEFR% (OR: 3.2, p<0.00001) and FEV1% (OR: 12.8, p<0.00001). Notably, abnormal FVC% was most strongly associated with RA (OR: 26.4, p<0.00001), indicating a marked impact on lung function.

## Discussion:

This study demonstrates a significant association between rheumatoid arthritis (RA) and pulmonary function abnormalities, particularly restrictive patterns, among Indian patients. A substantial proportion (44%) of RA patients exhibited abnormal FVC% (Table 1 - see PDF), suggesting restrictive lung involvement. This finding aligns with studies from Eastern India, where 44% showed restrictive abnormalities [6] and from North India, which reported 29% prevalence [7]. The strong association in our data is further reinforced by a high odds ratio for FVC% abnormality (OR = 26.4, p < 0.00001, Table 5 - see PDF), indicating a strong link between RA and restrictive pulmonary disease, likely due to interstitial lung involvement [8]. In contrast, only 2% of patients showed obstructive abnormalities based on the FEV1/FVC ratio. This is lower than the 6.6% prevalence of large airway obstruction reported by Praba *et al.* (2017) [9]. Such discrepancies may result from regional differences in smoking habits, environmental exposure, or genetic predisposition. Furthermore, 67% of our patients had high DAS-28 scores (Table 4 - see PDF), which is associated with worse pulmonary function. This finding supports similar results from Iran, where elevated DAS-28 scores were linked to pulmonary function abnormalities [8]. Duration of RA also appeared to influence pulmonary involvement. Sixty percent of the patients had RA for 3-4 years (Table 3 - see PDF) and longer disease duration showed a strong association with restrictive lung patterns (p < 0.00001). This pattern is comparable to findings in Karnataka, where 40% of patients with over five years of disease developed pulmonary complications [10]. All patients in this study were seropositive for either rheumatoid factor (RF) or anti-cyclic citrullinated peptide antibodies (ACPA) (Table 2 - see PDF), consistent with Eastern Indian studies that reported a strong correlation between seropositivity and pulmonary involvement [6]. Notably, 28% of patients had high ACPA levels, which may exacerbate lung inflammation through deposition of citrullinated proteins [7]. In addition, 67% showed elevated CRP levels, which supports findings from Tamil Nadu that link systemic inflammation to progressive pulmonary decline [6]. Although the restrictive abnormality rate in this study was 44%, lower rates have been reported in other regions, such as 10% prevalence in Madhya Pradesh [9], possibly due to variation in diagnostic criteria or genetic diversity. The observed female predominance (82% in RA cases, Table 1 - see PDF) reflects national trends. However, the severity of pulmonary dysfunction was more pronounced in this cohort compared to similar South Indian studies [11], suggesting that environmental exposures or comorbid conditions may contribute to the observed differences.

## Conclusion:

A strong association between rheumatoid arthritis and abnormal pulmonary function, with restrictive patterns being most common is shown. High disease activity, longer RA duration and elevated serological markers were linked to significant PFT abnormalities. Routine pulmonary screening should be integrated into RA management to enable early detection and improve outcomes.
